# The SH3 and cysteine-rich domain 3 (*Stac3*) gene is important to growth, fiber composition, and calcium release from the sarcoplasmic reticulum in postnatal skeletal muscle

**DOI:** 10.1186/s13395-016-0088-4

**Published:** 2016-04-11

**Authors:** Xiaofei Cong, Jonathan Doering, Davi A. G. Mazala, Eva R. Chin, Robert W. Grange, Honglin Jiang

**Affiliations:** Department of Animal and Poultry Sciences, Virginia Tech, Blacksburg, VA USA; Department of Human Nutrition, Foods, and Exercise, Virginia Tech, Blacksburg, VA USA; Department of Kinesiology, School of Public Health, University of Maryland, College Park, MD USA

**Keywords:** Skeletal muscle, Hypertrophy, Fiber type, Calcium release

## Abstract

**Background:**

The SH3 and cysteine-rich domain 3 (*Stac3*) gene is specifically expressed in the skeletal muscle. *Stac3* knockout mice die perinatally. In this study, we determined the potential role of *Stac3* in postnatal skeletal muscle growth, fiber composition, and contraction by generating conditional *Stac3* knockout mice.

**Methods:**

We disrupted the *Stac3* gene in 4-week-old male mice using the Flp-FRT and tamoxifen-inducible Cre-loxP systems.

**Results:**

RT-qPCR and western blotting analyses of the limb muscles of target mice indicated that nearly all *Stac3* mRNA and more than 70 % of STAC3 protein were deleted 4 weeks after tamoxifen injection. Postnatal *Stac3* deletion inhibited body and limb muscle mass gains. Histological staining and gene expression analyses revealed that postnatal *Stac3* deletion decreased the size of myofibers and increased the percentage of myofibers containing centralized nuclei, with no effect on the total myofiber number. Grip strength and grip time tests indicated that postnatal *Stac3* deletion decreased limb muscle strength in mice. Muscle contractile tests revealed that postnatal *Stac3* deletion reduced electrostimulation-induced but not the ryanodine receptor agonist caffeine-induced maximal force output in the limb muscles. Calcium imaging analysis of single flexor digitorum brevis myofibers indicated that postnatal *Stac3* deletion reduced electrostimulation- but not caffeine-induced calcium release from the sarcoplasmic reticulum.

**Conclusions:**

This study demonstrates that STAC3 is important to myofiber hypertrophy, myofiber-type composition, contraction, and excitation-induced calcium release from the sarcoplasmic reticulum in the postnatal skeletal muscle.

**Electronic supplementary material:**

The online version of this article (doi:10.1186/s13395-016-0088-4) contains supplementary material, which is available to authorized users.

## Background

The *Stac3* gene, which encodes a protein containing Src homology 3 and cysteine-rich domains, is expressed specifically in the skeletal muscle [1]. STAC3 has been recently identified as a novel regulator of embryonic skeletal muscle development and contraction. *Stac3* knockout mice die at birth [1, 2]. Most of the muscle fibers in newborn *Stac3* knockout mice contain centralized nuclei and disorganized myofibrils [1].

The skeletal muscles from *Stac3*-deleted mouse fetuses do not contract, and this is suggested to be due to defective excitation-contraction (EC) coupling [2]. A role of STAC3 in EC coupling is further supported by immunoprecipitation data from zebrafish that suggests STAC3 interacts with the dihydropyridine receptors (DHPR) and the ryanodine receptors (RYR) [3] and immunohistochemical data from mice that indicates STAC3 co-localizes with DHPR at the T-tubules [2]. A more recent study demonstrates that STAC3 is essential for trafficking of the major subunit of DHPR, DHPRα1s, also known as Cav1.1 and CACNA1S, into cell membranes [4]. In vitro studies suggest that STAC3 might be involved in myoblast differentiation [5, 6]. Mutations in the human *Stac3* gene have been linked to two congenital myopathies, Native American myopathy [3] and King-Denborough syndrome [7].

In this study, we determined the potential role of STAC3 in postnatal skeletal muscle growth, fiber composition, and contraction. We disrupted the *Stac3* gene in 4-week-old mice through the Flp-FRT and Cre-loxP systems [8, 9]. Our study demonstrates that STAC3 is important to fiber hypertrophy, fiber-type composition, muscle contraction, and electrostimulation-induced calcium release from the sarcoplasmic reticulum in the postnatal skeletal muscle.

## Methods

### Generation of conditional *Stac3* knockout mice

The generation of heterozygous *Stac3* mutant mice (Stac3^+/−)^ has been described [1]. The mutant *Stac3* allele was inserted with a trapping cassette (SA-βgeo-pA) flanked by two flippase (Flp) recombinase target sites (flippase recognition target, FRT) between exons 1 and 2 and two Cre recombinase target sites (loxP) that flanked exons 2 to 5. Transgenic mice that expressed a Flp recombinase (*Tg*^*Flp*^) driven by the human beta actin promoter [B6.Cg-Tg(ACTFLPe)9205Dym/J] and transgenic mice (*Tg*^*Cre*^) that carried a tamoxifen-inducible Cre recombinase driven by the chicken beta actin promoter/enhancer coupled with the cytomegalovirus immediate-early enhancer [B6.Cg-Tg(CAG-cre/Esr1*)5Amc/J] were purchased from the Jackson Laboratory (Bar Harbor, ME) [10]. When purchased, these transgenic mice had been backcrossed onto the C57BL/6J background for at least five generations. A *Stac3*^*+/−*^ mouse was mated to a *Tg*^*Flp*^ mouse to generate offspring (*Stac3*^*+/fl*^) in which the mutant *Stac3* allele was converted to a pre-conditioned wild-type allele. Two *Stac3*^*+/fl*^ mice were mated to generate *Stac3*^*fl/fl*^ mice in which both *Stac3* alleles were converted to the pre-conditioned alleles. A *Stac3*^*fl/fl*^ mouse was crossed with a *Tg*^*Cre*^ mouse to generate *Stac3*^*+/fl*^*Tg*^*Cre*^ mice. One *Stac3*^*+/fl*^*Tg*^*Cre*^ mouse and one *Stac3*^*fl/fl*^ mouse were mated to generate *Stac3*^*fl/fl*^*Tg*^*Cre*^ mice. Male *Stac3*^*fl/fl*^, *Stac3*^*+/fl*^*Tg*^*Cre*^, and *Stac3*^*fl/fl*^*Tg*^*Cre*^ mice at 4 weeks of age were injected intraperitoneally with tamoxifen (Sigma-Aldrich, St. Louis, MO) dissolved in corn oil at a daily dose of 75 mg/kg body mass for five consecutive days to activate the transcription of the *Cre* transgene and hence to delete the *Stac3*^*fl*^ allele. All mice were kept on a 12-h light/12-h dark cycle at 23 °C. Mice had ad libitum access to food (rodent diet 2918, Harlan, Indianapolis, IN) and water. All protocols involving mice were approved by the Virginia Tech Institutional Animals Care and Use Committee.

### Genotyping

Mice were genotyped by PCR of genomic DNA isolated from ear notches followed by gel electrophoresis. Genomic DNA was isolated using the DNeasy Blood & Tissue Kit (Qiagen, Hilden, Germany). Initial PCR products were confirmed by DNA sequencing. The *Flp* and *Cre* transgenes were identified using primer pairs suggested by the Jackson Laboratory. The sizes of PCR products from these two pairs of primers were expected to be 725 and 100 bp, respectively. The wild-type and trapped *Stac3* alleles were identified by PCR using primers F1 and R1. These two primers amplified a 317-bp product from the wild-type *Stac3* allele and a 344-bp product from the trapped *Stac3* allele. The Flp-cleaved *Stac3* allele (*Stac3*^*fl*^) was confirmed by PCR using primers F2 and R2. These two primers were designed to flank the trapping cassette to generate a 451-bp product from the *Stac3*^*fl*^ allele, a 259-bp product from the wild-type *Stac3* allele, and no product from the trapped *Stac3* allele. The Cre-recombined *Stac3*^*fl*^ allele (*Stac3*^*flΔ*^) was confirmed by PCR using primers F2 and R1, which amplified a 2478-bp product from the *Stac3*^*fl*^ allele, a 2259-bp product from the wild-type *Stac3* allele, and a 530-bp product unique to the *Stac3*^*flΔ*^ allele. Sequences of all primers used in this study are presented in Additional file 1: Table S1.

### Reverse transcription-quantitative polymerase chain reaction (RT-qPCR)

Total RNA from mouse tissue samples was extracted using the TRI reagent (Molecular Research Center, Inc., Cincinnati, OH), following the manufacturer’s instructions. Concentrations of RNA samples were determined using a NanoDrop 2000 Spectrophotometer (Thermo Fisher Scientific, Pittsburgh, PA). First-strand cDNA synthesis was performed using random primers and ImProm-II reverse transcriptase (Promega Corp., Madison, WI). Quantitative PCR was performed using SyberGreen PCR Master Mix (Life Technologies Corp., Carlsbad, CA) and gene-specific primers (Additional file 1: Table S1) on a CFX96 Touch Real-Time PCR Detection System (Bio-Rad Laboratories, Inc., Hercules, CA). Primers were designed using the Primer3 software (MIT, Cambridge, MA). The specificity of all primers was verified by agarose gel electrophoresis followed by DNA sequencing. The amplification efficiency of all primers was determined by analyzing the standard curve of serially diluted cDNA. The amplification efficiency of the primers used in this study ranged from 90 to 110 %. The relative abundance of a mRNA to *18S* rRNA was calculated using the ΔΔCt method [11].

### Western blotting analysis

Mouse muscle samples were lysed in radio immunoprecipitation assay (RIPA) buffer consisting of 25 mM Tris-HCl, 150 mM NaCl, 1 % NP-40, 1 % sodium deoxycholate, 0.1 % SDS, and 1 % protease inhibitors (Thermo Fisher Scientific) at 4 °C for 30 min. The lysates were centrifuged at 12,000×*g* for 15 min at 4 °C. Protein concentrations in the supernatants were measured using a BCA Protein Assay Kit (Thermo Fisher Scientific). For western blotting, 30 μg of total cellular protein were resolved by 10 % (for STAC3) or 6 % (for CACNA1S and RYR1) SDS-PAGE followed by transfer to a 0.2 μm nitrocellulose membrane (Bio-Rad Laboratories). The membrane was blocked with 5 % non-fat milk in TBST (20 mM Tris-HCl, 500 mM NaCl, and 0.1 % Tween-20) at room temperature for 1 h and then incubated with 1:1000 diluted primary antibody at 4 °C overnight. The membrane was then incubated with 1:20,000 diluted secondary antibody and visualized with the ODYSSEY CLx system (LI-COR Biosciences, Lincoln, NE). Following the detection of the primary target protein, the membrane was stripped in Restore Western Blot Stripping Buffer (Thermo Fisher Scientific) and re-probed with the antibody for beta tubulin (TUBB). The following primary antibodies were used: CACNA1S (1A, Thermo Fisher Scientific), RYR1 (34C, DHSB, Iowa City, IA), STAC3 (Proteintech Group, Inc., Chicago, IL), and TUBB (E7, DHSB). The following secondary antibodies were used: IRDye goat anti-mouse 800CW and IRDye goat anti-rabbit 800CW (LI-COR).

### Hematoxylin and eosin staining

The extensor digitorum longus (EDL) and soleus (SOL) muscles from the right hindlimbs of mice were characterized. Following dissection, a 5-mm-long segment was cut from the middle of each muscle, immersed in optimal cutting temperature compound on a cork sheet, and frozen immediately in isopentane pre-cooled in liquid nitrogen. The samples were then left in a Leica CM 1800 cryostat at −18 °C for 20 min for thermal adaptation. Cross-sections of 8 μm were cut and mounted on Fisher Superfrost Plus slides (Thermo Fisher Scientific) for subsequent assays. Hematoxylin and eosin staining was performed as described previously [1]. Stained sections were imaged with an Eclipse TS100 microscope (Nikon Corp., Tokyo, Japan).

### Myosin-ATPase and nicotinamide adenine dinucleotide-tetrazolium reductase (NADH-TR) staining

Myosin-ATPase and NADH-TR staining of the EDL and SOL sections prepared above were conducted as described previously [12]. Briefly, for myosin-ATPase staining, muscle sections were pre-incubated for 5 min in a sodium barbital buffer containing 20 mM sodium barbital and 36 mM CaCl_2_ at pH 4.21 and then incubated for 55 min in a sodium barbital buffer containing 3.5 mM ATP, 20 mM sodium barbital, and 18 mM CaCl_2_ at pH 9.45. The sections were rinsed in 1 % CaCl_2_ for 3 × 1 min and then immersed in 2 % CoCl_2_ for 2 min. The sections were then stained with 1 % (NH_4_)_2_S for 20 s followed by five rinses with ddH_2_O. Stained sections were dehydrated in 50, 70, 80, 90, and 100 % ethanol, cleared in xylene, and then mounted in 50 % xylene and 50 % Canada balsam (Thermo Fisher Scientific) mixture. The activity of NADH-TR in the muscle sections was determined by incubating the sections at 37 °C for 10 min and then with 1 mg/ml nitroblue tetrazolium and 0.4 mg/ml β-NADH (Sigma-Aldrich) in 50 mM Tris-HCl buffer, pH 7.3, at 37 °C for 30 min. After 5 × 5 min washes in a graded acetone series (30, 60, 90, 60, and 30 %), the sections were washed with ddH_2_O, mounted in prolong gold mounting medium (Invitrogen), cover-slipped, and imaged with an Eclipse TS100 microscope (Nikon Corp.).

### Grip strength and grip time tests

Grip strength and grip time were measured weekly at approximately 9:00 a.m. Grip strength was tested using a digital mouse grip strength meter (AMETEK, Inc. Berwyn, PA). The mouse was allowed to grip a wire mesh grid with its front paws and then was gently pulled away by its tail until it released the grid. This was repeated three times, and the mouse was allowed to rest for 5 min between two consecutive tests. The maximum force (mN) generated by each pull was recorded by a force transducer attached to the grid, and values from three pulls were averaged for each mouse. Grip time was determined using a coarse metal wire with a 2 mm diameter. The mouse was allowed to hold the wire using its front paws. The time from initial grip to release was recorded as grip time. Data from tests in which the mouse was unwilling to hold the wire and dropped off consciously were discarded. The grip time test was repeated at least three times with 5 min of rest between two consecutive tests. Grip time from three successful tests was averaged for each mouse. The mouse’s motivational or cardiovascular status could affect the measured grip strength or time, and these statuses were not evaluated in these tests.

### Muscle contractile tests

Fast-twitch EDL and slow-twitch SOL muscles were excised from euthanized mice at 8 weeks of age, i.e., 4 weeks after tamoxifen injection. The isolated muscles were incubated at 30 °C in an oxygenated (95 % O_2_ and 5 % CO_2_) physiological salt solution (PSS) as previously described [13]. Non-absorbable braided silk suture (4-0) was tied to the distal and proximal tendons at the myotendinous junctions. Muscles were then fixed between a clamp and the arm of a Dual-Mode Servomotor System (300B, Aurora Scientific Inc., Aurora, Ontario, Canada) at a resting tension of 1.0 g which was maintained by a stepper motor. One EDL muscle and one SOL muscle from the same mouse were tested under the same treatment conditions at the same time. The servomotor arm and stepper motor were controlled by Dynamic Muscle Control software (DMC Version 4.1.6, Aurora Scientific) to obtain the tension output data.

The stimulated muscle protocol consisted of three steps. In step 1, the stimulated muscles were subjected to three isometric twitches and tetani (150 Hz) spaced 1 min apart. In step 2, after a 10-min quiescent period, the muscles were subjected to a force-frequency protocol at electrical stimulation frequencies of 1, 30, 50, 80, 100, and 150 Hz. Stimulation frequencies were spaced 1 min apart and were delivered as 200-μs square wave pulses, at 20 V. In step 3, after 5 min of rest, the stepper motor was turned off, the PSS-only buffer drained, and fresh PSS buffer containing 25 mM caffeine was added to the EDL and SOL muscle baths. At the end of each experiment, after a 10-min washout with PSS, each muscle was gently blotted dry on a Kimwipe; muscle mass was determined to the nearest 0.1 mg using an A-200D electronic analytical balance (Denver Instruments, Denver, CO).

Tension output profiles were analyzed using the Dynamic Muscle Analysis software (DMA Version 3.2, Aurora Scientific) to determine contractile properties, including peak tension, time to peak tension (TPT), and half-relaxation time (HRT). Muscle cross-sectional area (CSA) was determined as previously described [14]. Twitch and tetanic tensions were normalized to muscle CSA to obtain twitch and tetanic stress (mN/mm^2^).

### Single myofiber isolation

Flexor digitorum brevis (FDB) muscles were isolated from 8-week-old mice and digested in minimum essential medium (MEM) solution containing 10 % fetal bovine serum (FBS), 1 % penicillin-streptomycin, and 0.2 % collagenase type 2 (Worthington Biochemical Corp., Lakewood, NJ) for 4 h at 37 °C in a tissue culture incubator (5 % CO_2_). The muscle samples were then gently triturated with plastic Pasteur pipettes into a 24-well plate with MEM solution containing 10 % FBS and 1 % penicillin-streptomycin. Single muscle fibers were obtained by trituration. Subsequently, fibers were maintained in MEM solution with 10 % FBS at 37 °C until used for EC coupling assessment.

### EC coupling assessment in single myofibers

Single muscle fibers were loaded with Fura-4F AM for 15 min. The Fura-4F ratio was measured in response to electrical stimulation as an index of [Ca^2+^]_i_. Fibers loaded with Fura-4F were placed in a stimulation chamber containing parallel electrodes and the chamber was positioned on a Nikon TiU microscope stage. Muscle fibers were continuously perfused with a stimulating Tyrode solution (121.0 mM NaCl, 5.0 mM KCl, 1.8 mM CaCl_2_, 0.5 mM MgCl_2_, 0.4 mM NaH_2_PO_4_, 24.0 mM NaHCO_3_, and 5.5 mM glucose) with 0.2 % FBS [15]. This solution was bubbled with 95 % O_2_ and 5 % CO_2_ to maintain a pH of 7.3 [15]. Levels of [Ca^2+^]_i_ were assessed by the Fura-4F fluorescence ratio using an IonOptix HyperSwitch system with a dual excitation, single emission filter set for Fura-4F (excitation 340 and 380 nm; emission 510 nm). Signals were captured and analyzed using the IonWizard software (IonOptix). Global Fura-4F ratio was measured in the muscle fibers using trains of stimuli at 10, 30, 50, 70, and 100 Hz for 350 ms with fibers resting 1 min between frequencies. Peak Fura-4F ratios at each frequency were determined by the average ratio in the last 100 ms of the 350-ms tetanus when Ca^2+^ Fura-4F should be at a steady state. All single muscle fibers were evaluated at room temperature.

### Statistics

Student’s *t* test was used to determine the statistical significance of the differences between two groups. ANOVA followed by Tukey’s test was used to examine the statistical significance of differences between more than two groups. A difference was considered statistically significant when *P* < 0.05. All data were presented as mean ± standard error of the mean (SEM).

## Results

### Postnatal deletion of the *Stac3* gene in mice

**Fig. 1:**
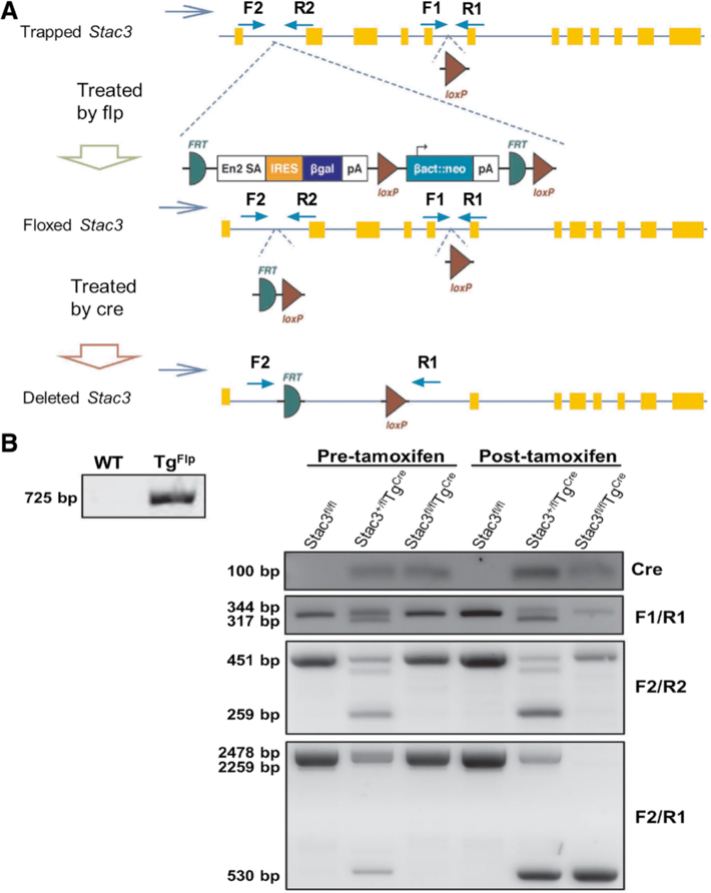
Generation of conditional *Stac3* knockout mice. **a** Schematic representation of the process of generating conditional *Stac3* knockout mice. The original mutant *Stac3* allele contained a “trapping cassette” between *Stac3* exons 1 and 2 and a loxP site between exons 5 and 6. Crossing heterozygous *Stac3* mutant mice with mice expressing the Flp recombinase caused the trapping cassette to be deleted at the two FRT sites and hence converted the mutant *Stac3* allele to a floxed allele, i.e., a pre-conditional wild-type allele. Crossing mice bearing the floxed *Stac3* allele with mice expressing the Cre recombinase caused exons 2 to 5 flanked by the loxP sites to be deleted and hence inactivated the *Stac3* gene. In the Cre recombinase transgenic mice used in this study, the *Cre* gene is located downstream of a tamoxifen-inducible chicken beta actin promotor. *Arrows* labeled with *F1*, *R1*, *F2*, and *R2* indicate the locations of PCR primers for genotyping. **b** Representative gel images of genotyping. Genotypes of *Stac3* ^*fl/fl*^, *Stac3* ^*+/fl*^ *Tg* ^*Cre*^, and *Stac3* ^*fl/fl*^ *Tg* ^*Cre*^ mice before and 4 weeks after tamoxifen injection were identified by PCR of genomic DNA followed by gel electrophoresis. Names of PCR primers or target genes are indicated on the right of gel images and sizes of expected PCR products on the left of gel images. *WT* wild type

The original mutant *Stac3* allele contained two Frt sites between exons 1 and 2 and two loxP sites that flanked exons 2 and 5 (Fig. 1a). By crossing *Stac3*^*+/−*^ mice first with mice expressing the Flp recombinase (*Tg*^*Flp*^) and then mice expressing the tamoxifen-inducible Cre recombinase (*Tg*^*Cre*^), we generated mice with the following genotypes: *Stac3*^*fl/fl*^*Tg*^*Cre*^, which were homozygous for the floxed *Stac3* allele and carried the *Cre* recombinase transgene; *Stac3*^*+/fl*^*Tg*^*Cre*^, which were heterozygous for the floxed *Stac3* allele and carried the *Cre* recombinase transgene; and *Stac3*^*fl/fl*^, which were homozygous for the floxed *Stac3* allele but did not carry the *Cre* recombinase transgene. We confirmed these genotypes by PCR of genomic DNA from these mice (Fig. 1a, b).

**Fig. 2:**
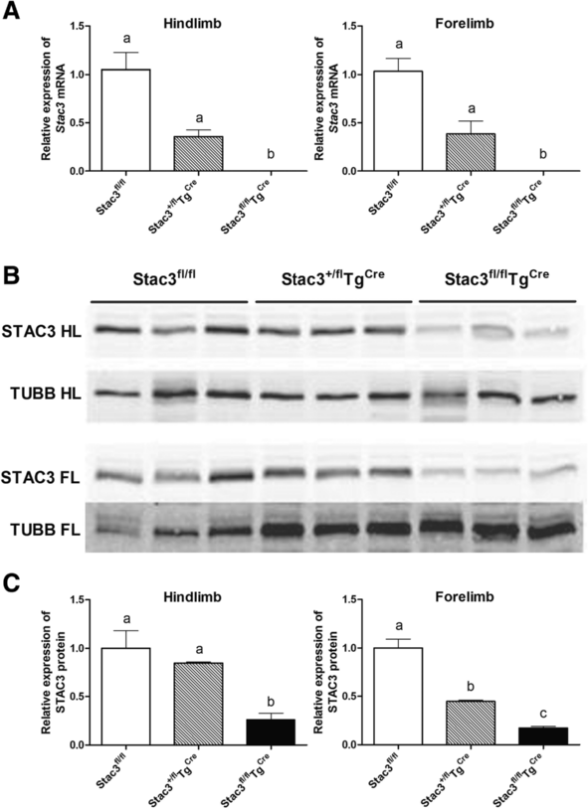
Validation of *Stac3* knockout. **a** Analysis of *Stac3* mRNA expression. *Stac3* mRNA in the hindlimb and forelimb muscles of *Stac3* ^*fl/fl*^, *Stac3* ^*+/fl*^ *Tg* ^*Cre*^, and *Stac3* ^*fl/fl*^ *Tg* ^*Cre*^ mice at 8 weeks of age, i.e., 4 weeks after tamoxifen injection, was quantified by RT-qPCR. *Bars* not sharing the same *letter labels* are different (*P* < 0.05, *n* = 4 mice). **b** Images from western blotting analyses of STAC3 protein in hindlimb (*HL*) and forelimb (*FL*) skeletal muscles of 8-week-old *Stac3* ^*fl/fl*^, *Stac3* ^*+/fl*^ *Tg* ^*Cre*^, and *Stac3* ^*fl/fl*^ *Tg* ^*Cre*^ mice. β-tubulin (TUBB) protein was used as a loading control. **c** Quantification of STAC3 protein band intensity. The expression level of STAC3 protein is normalized to that of β-tubulin in the same sample. *Bars* not sharing the same *letter labels* are different (*P* < 0.05, *n* = 3 mice)

We injected male *Stac3*^*fl/fl*^, *Stac3*^*+/fl*^*Tg*^*Cre*^, and *Stac3*^*fl/fl*^*Tg*^*Cre*^ mice at 4 weeks of age with tamoxifen for five consecutive days to induce the expression of Cre recombinase (Fig. 1a). RT-qPCR analyses of the hindlimb and forelimb muscles from these mice at 8 weeks of age, i.e., 4 weeks after tamoxifen injection, revealed that *Stac3* mRNA was nearly absent in *Stac3*^*fl/fl*^*Tg*^*Cre*^ mice compared to *Stac3*^*fl/fl*^ mice (Fig. 2a, *P* < 0.05). *Stac3* mRNA expression in 8-week-old *Stac3*^*+/fl*^*Tg*^*Cre*^ mice was reduced by 50 % compared to that in 8-week-old *Stac3*^*fl/fl*^ mice, but the difference was not statistically significant (Fig. 2a). Western blotting analyses revealed that STAC3 protein expression in hindlimb and forelimb muscles was reduced by more than 70 % in 8-week-old *Stac3*^*fl/fl*^*Tg*^*Cre*^ mice compared to 8-week-old *Stac3*^*fl/fl*^ mice (Fig. 2b c, *P* < 0.05). STAC3 protein expression in the forelimb muscles of 8-week-old *Stac3*^*+/fl*^*Tg*^*Cre*^ mice was reduced by approximately 50 % compared to that in 8-week-old *Stac3*^*fl/fl*^ mice (Fig. 2b, c, *P* < 0.05). STAC3 protein expression in the hindlimb muscles was not different between 8-week-old *Stac3*^*+/fl*^*Tg*^*Cre*^ and *Stac3*^*fl/fl*^ mice (Fig. 2b, c). Overall, these analyses demonstrated that nearly all *Stac3* mRNA and the majority of STAC3 protein were ablated by tamoxifen-induced Cre recombinase in *Stac3*^*fl/fl*^*Tg*^*Cre*^ mice.

### Postnatal *Stac3* deletion reduced body and muscle growth

**Fig. 3:**
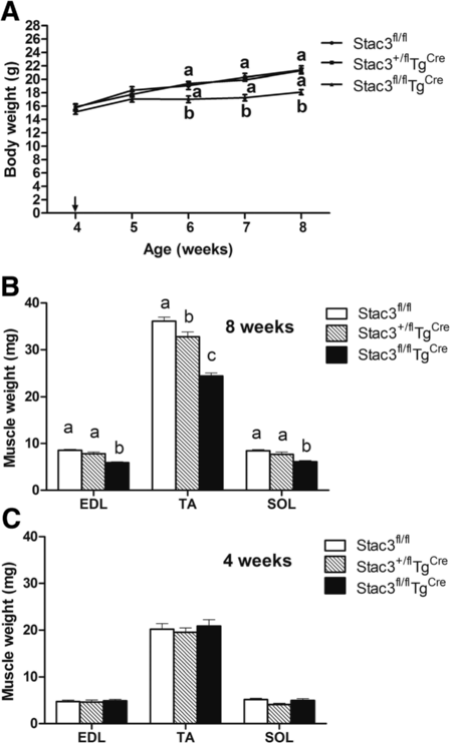
Body and muscle masses of *Stac3* ^*fl/fl*^, *Stac3* ^*+/fl*^ *Tg* ^*Cre*^, and *Stac3* ^*fl/fl*^ *Tg* ^*Cre*^ mice. **a** Body mass before and after tamoxifen injection. Tamoxifen injection was initiated on the first day of week 5 of age (indicated by a *black arrow*) and continued for 5 days. Body masses not sharing the same *letter labels* are different at the same age (*P* < 0.05, *n* = 4 mice). **b** Masses of EDL, TA, and SOL muscles at 8 weeks of age. *Bars* not sharing the same *letter labels* are different (*P* < 0.05, *n* = 4 mice). **c** Masses of EDL, TA, and SOL muscles at 4 weeks of age. The masses of these muscles were not different between 4-week-old *Stac3* ^*fl/fl*^, *Stac3* ^*+/fl*^ *Tg* ^*Cre*^, and *Stac3* ^*fl/fl*^ *Tg* ^*Cre*^ mice. *EDL* extensor digitorum longus, *TA* tibialis anterior, *SOL* soleus

From 4 to 8 weeks of age, *Stac3*^*fl/fl*^*Tg*^*Cre*^ mice gained approximately 2 g in body mass while control mice (*Stac3*^*+/fl*^*Tg*^*Cre*^ and *Stac3*^*fl/fl*^) gained approximately 5 g (Fig. 3a). The masses of the EDL, tibialis anterior (TA), and SOL muscles from 8-week-old *Stac3*^*fl/fl*^*Tg*^*Cre*^ mice were nearly 30 % less than those from 8-week-old *Stac3*^*+/fl*^*Tg*^*Cre*^ or *Stac3*^*fl/fl*^ mice (Fig. 3b, *P* < 0.05). The masses of the heart and liver were not different between 8-week-old *Stac3*^*fl/fl*^*Tg*^*Cre*^ mice and *Stac3*^*+/fl*^*Tg*^*Cre*^ or *Stac3*^*fl/fl*^ mice (data not shown). Neither body mass (Fig. 3a) nor individual muscle masses (Fig. 3b) was different between 4-week-old *Stac3*^*fl/fl*^*Tg*^*Cre*^ and *Stac3*^*+/fl*^*Tg*^*Cre*^ or *Stac3*^*fl/fl*^ mice. These data ruled out the possibility that differences in body and muscle masses between these mice at 8 weeks of age were due to differences in their initial genotypes.

### Postnatal *Stac3* deletion decreased muscle size and increased the number of myofibers with centralized nuclei

**Fig. 4:**
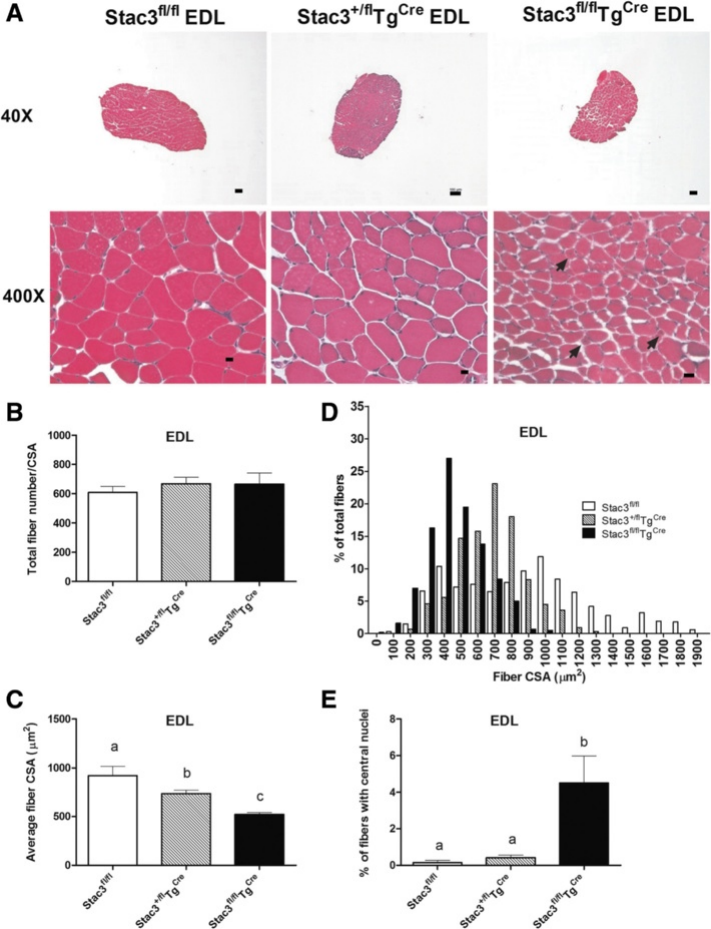
Histological analysis of EDL muscles from 8-week-old *Stac3* ^*fl/fl*^, *Stac3* ^*+/fl*^ *Tg* ^*Cre*^, and *Stac3* ^*fl/fl*^ *Tg* ^*Cre*^ mice. Cross-sections of EDL muscles were stained with hematoxylin and eosin. **a** Representative images of stained EDL sections at ×40 and ×400 magnifications. *Scale bars*: 100 μm for ×40 and 10 μm for ×400. *Arrows* point to myofibers with centralized nuclei. **b** Average number of myofibers per cross-section. This number was not different between genotypes. **c** Average cross-sectional area (CSA) of myofibers. **d** Distribution of myofiber CSA. **e** Percentage of myofibers containing centralized nuclei. *Bars* not sharing the same *letter labels* are different (*P* < 0.05, *n* = 4 mice)

**Fig. 5:**
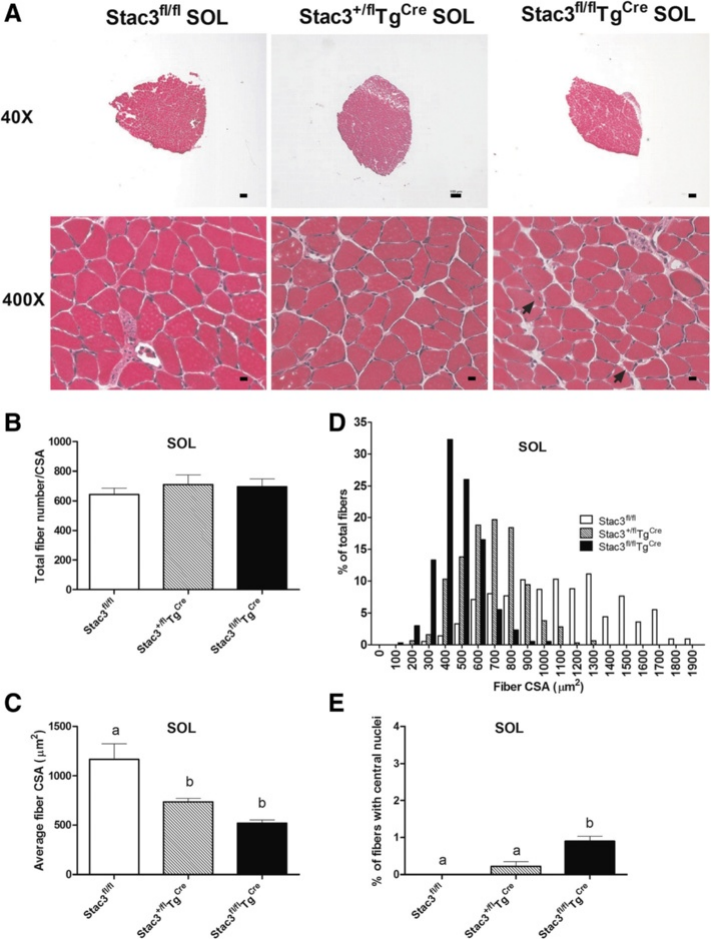
Histological analysis of SOL muscles from 8-week-old *Stac3* ^*fl/fl*^, *Stac3* ^*+/fl*^ *Tg* ^*Cre*^, and *Stac3* ^*fl/fl*^ *Tg* ^*Cre*^ mice. Cross-sections of SOL muscles were stained with hematoxylin and eosin. **a** Representative images of stained SOL sections at ×40 and ×400 magnifications. *Scale bars*: 100 μm for ×40 and 10 μm for ×400. *Arrows* point to myofibers with centrally located nuclei. **b** Average number of myofibers per cross-section. This number was not different between genotypes. **c** Average CSA of myofibers. **d** Distribution of CSA of myofibers. **e** Percentage of myofibers containing centralized nuclei. *Bars* not sharing the same *letter labels* are different (*P* < 0.05, *n* = 4 mice)

Decreased muscle size and the presence of centralized nuclei were evident in the *Stac3*^*fl/fl*^*Tg*^*Cre*^ EDL compared to the other genotypes (Fig. 4a). Total myofiber numbers in EDL muscles of 8-week-old *Stac3*^*fl/fl*^, *Stac3*^*+/fl*^*Tg*^*Cre*^, and *Stac3*^*fl/fl*^*Tg*^*Cre*^ mice did not differ (Fig. 4b). The average CSA of EDL myofibers in 8-week-old *Stac3*^*fl/fl*^*Tg*^*Cre*^ mice was smaller than that in 8-week-old *Stac3*^*+/fl*^*Tg*^*Cre*^ mice, and the latter was smaller than that in 8-week-old *Stac3*^*fl/fl*^ mice (Fig. 4c, *P* < 0.05). The distribution of CSA of 8-week-old *Stac3*^*fl/fl*^*Tg*^*Cre*^ EDL myofibers was skewed to the left compared to that of 8-week-old *Stac3*^*+/fl*^*Tg*^*Cre*^ or *Stac3*^*fl/fl*^ EDL myofibers (Fig. 4d). The EDL muscle in 8-week-old *Stac3*^*fl/fl*^*Tg*^*Cre*^ mice contained more myofibers with centralized nuclei than that in *Stac3*^*+/fl*^*Tg*^*Cre*^ or *Stac3*^*fl/fl*^ mice (Fig. 4e, *P* < 0.05). Similar differences in myofiber CSA and number of myofibers with centralized nuclei were observed for the SOL muscles between *Stac3*^*fl/fl*^*Tg*^*Cre*^ and *Stac3*^*fl/fl*^ or *Stac3*^*+/fl*^*Tg*^*Cre*^ mice (Fig. 5a–e). However, none of these differences were observed for the EDL or SOL muscles from *Stac3*^*fl/fl*^, *Stac3*^*+/fl*^*Tg*^*Cre*^, and *Stac3*^*fl/fl*^*Tg*^*Cre*^ mice at 4 weeks of age, i.e., before tamoxifen injection (Additional file 2: Figure S1 and Additional file 3: Figure S2).

### Postnatal *Stac3* deletion increased the percentage of type I and oxidative myofibers

**Fig. 6:**
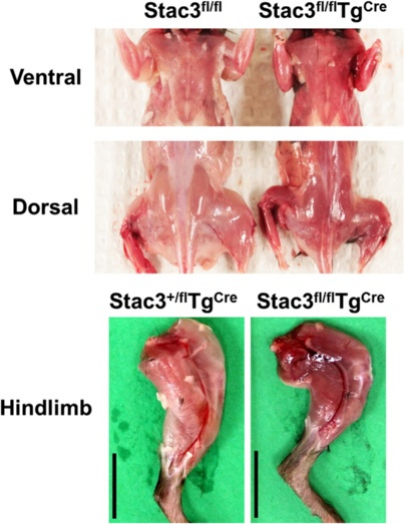
Representative images of body and limb muscles of 8-week-old *Stac3* ^*fl/fl*^ and *Stac3* ^*fl/fl*^ *Tg* ^*Cre*^ mice immediately after euthanasia. Note that the *Stac3* ^*fl/fl*^ *Tg* ^*Cre*^ skeletal muscles appeared redder

**Fig. 7:**
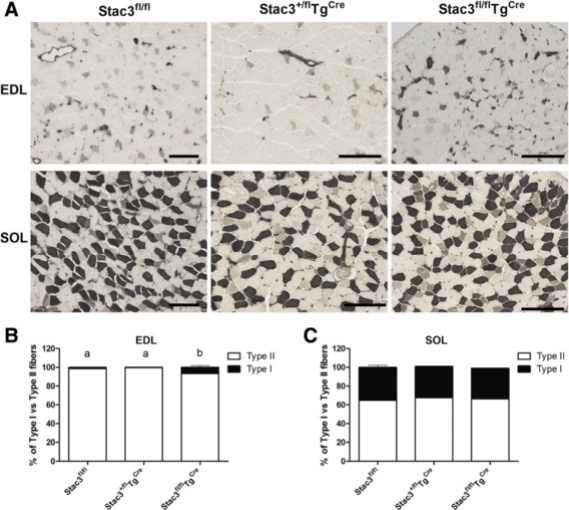
Myosin-ATPase staining (pH 4.21) of EDL and SOL muscle sections from 8-week-old *Stac3* ^*fl/fl*^, *Stac3* ^*+/fl*^ *Tg* ^*Cre*^, and *Stac3* ^*fl/fl*^ *Tg* ^*Cre*^ mice. **a** Representative images of stained muscle sections. *Scale bars*: 100 μm. **b** Percentages of type I and type II myofibers in the EDL muscle. **c** Percentages of type I and type II myofibers in the SOL muscle. Values not sharing the same *letter labels* are different (*P* < 0.05, *n* = 4 mice)

**Fig. 8:**
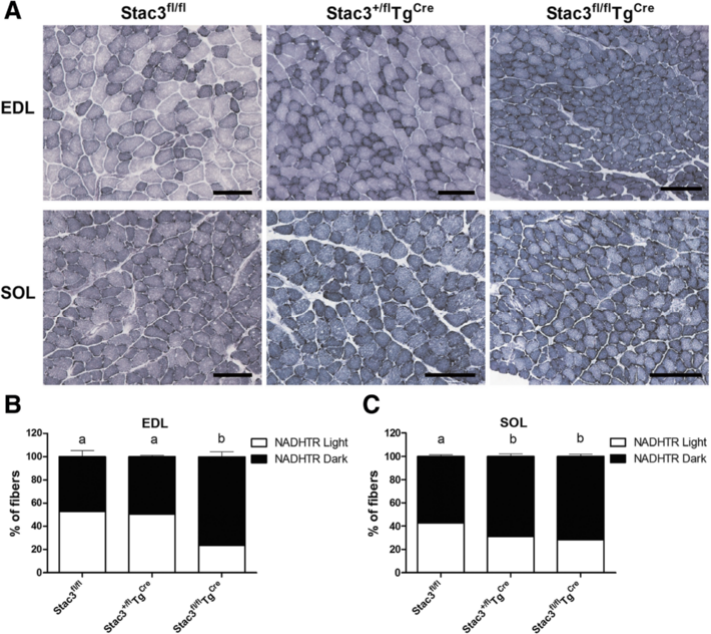
NADH-TR staining of EDL and SOL muscle sections from 8-week-old *Stac3* ^*fl/fl*^, *Stac3* ^*+/fl*^ *Tg* ^*Cre*^, and *Stac3* ^*fl/fl*^ *Tg* ^*Cre*^ mice. **a** Representative images of NADH-TR staining. *Scale bars*: 100 μm. **b** Percentages of light-stained and dark-stained myofibers in the EDL muscles. **c** Percentages of light-stained and dark-stained myofibers in the SOL muscles. Values not sharing the same *letter labels* are different (*P* < 0.05, *n* = 4 mice)

Body musculature of 8-week-old *Stac3*^*fl/fl*^*Tg*^*Cre*^ mice appeared to be redder than those from 8-week-old *Stac3*^*fl/fl*^ mice (Fig. 6). Based on myosin-ATPase staining at pH 4.21 (Fig. 7a), the 8-week-old *Stac3*^*fl/fl*^*Tg*^*Cre*^ EDL muscle contained a higher percentage of type I fibers and a lower percentage of type II (IIa, b, and x) fibers than the 8-week-old *Stac3*^*fl/fl*^ or *Stac3*^*+/fl*^*Tg*^*Cre*^ EDL muscle (Fig. 7b, *P* < 0.05). The percentage of type I or type II fibers was not different between the 8-week-old *Stac3*^*fl/fl*^ and *Stac3*^*+/fl*^*Tg*^*Cre*^ EDL muscles (Fig. 7b). The percentage of type I or type II myofibers was not different between the 8-week-old *Stac3*^*fl/fl*^*Tg*^*Cre*^ and *Stac3*^*fl/fl*^ or *Stac3*^*+/fl*^*Tg*^*Cre*^ SOL muscle (Fig. 7c). The percentage of type I or type II fibers in the EDL or the SOL muscle was not different between 4-week-old *Stac3*^*fl/fl*^*Tg*^*Cre*^ and *Stac3*^*fl/fl*^ or *Stac3*^*+/fl*^*Tg*^*Cre*^ mice (Additional file 4: Figure S3). Based on NADH-TR staining (Fig. 8a), the 8-week-old *Stac3*^*fl/fl*^*Tg*^*Cre*^ EDL and SOL muscles contained higher percentages of myofibers that stained darker, meaning greater oxidative capacity, than the 8-week-old *Stac3*^*fl/fl*^ or *Stac3*^*+/fl*^*Tg*^*Cre*^ EDL and SOL muscles (Fig. 8b, *P* < 0.05). Neither NADH-TR staining of the EDL muscle nor that of the SOL muscle was different between 4-week-old *Stac3*^*fl/fl*^*Tg*^*Cre*^ and *Stac3*^*fl/fl*^ or *Stac3*^*+/fl*^*Tg*^*Cre*^ mice (Additional file 5: Figure S4).

### Postnatal *Stac3* deletion increased the expression of genes marking type I fibers and developing muscle

We compared the expression levels of genes *Actn3*, *Myh1*, *Myh2*, *Myh4*, and *Tnnt3* that are markers of type II myofibers and the expression levels of genes *Mb*, *Mef2c*, *Myh7*, *Ppargc1a*, and *Tnnt1* that are markers of type I myofibers, in the hindlimb muscles of 8-week-old *Stac3*^*fl/fl*^*Tg*^*Cre*^, *Stac3*^*+/fl*^*Tg*^*Cre*^, and *Stac3*^*fl/fl*^ mice. As shown in Table 1, four of the five type I fiber marker genes including *Myh7*, which is the only myosin heavy chain gene expressed in type I fibers, were expressed at greater levels in 8-week-old *Stac3*^*fl/fl*^*Tg*^*Cre*^ than in 8-week-old *Stac3*^*fl/fl*^ or *Stac3*^*+/fl*^*Tg*^*Cre*^ mice; two of the five type II fiber marker genes including *Myh4*, the predominant myosin heavy chain expressed in type II fibers, were expressed at lower levels in 8-week-old *Stac3*^*fl/fl*^*Tg*^*Cre*^ than in 8-week-old *Stac3*^*fl/fl*^ or *Stac3*^*+/fl*^*Tg*^*Cre*^ mice. Among the five type II fiber marker genes analyzed, expression of *Actn3* mRNA was greater in 8-week-old *Stac3*^*fl/fl*^*Tg*^*Cre*^ than in 8-week-old *Stac3*^*fl/fl*^ or *Stac3*^*+/fl*^*Tg*^*Cre*^ mice (Table 1). The *Myh3* and *Myh8* genes are typically expressed in fetal and perinatal muscle or regenerating adult muscle [16, 17]. Both *Myh3* and *Myh8* mRNAs were expressed at greater levels in the 8-week-old *Stac3*^*fl/fl*^*Tg*^*Cre*^ than in the 8-week-old *Stac3*^*fl/fl*^ or *Stac3*^*+/fl*^*Tg*^*Cre*^ muscles (Table 1).

**Table 1:** Relative expression levels of selected mRNAs in left hindlimb muscles of mice 4 weeks after tamoxifen injection

Gene	Fiber type	*Stac3* ^*fl/fl*^	*Stac3* ^*+/fl*^ *Tg* ^*Cre*^	*Stac3* ^*fl/fl*^ *Tg* ^*Cre*^
*Actn3*	II	799.4 ± 232.6^a^	6040.1 ± 1281.6^b^	22679.4 ± 4486.4^c^
*Myh1*	II	569.0 ± 126.4^a^	747.1 ± 129.2^ab^	1472.3 ± 146.5^b^
*Myh2*	II	769.9 ± 188.9	1124.7 ± 163.5	1373.4 ± 140.1
*Myh4*	II	17463.2 ± 976.8^a^	11812.9 ± 1427.3^ab^	8553.5 ± 1702.5^b^
*Tnnt3*	II	104459.0 ± 32168.1^a^	42788.0 ± 7435.6^b^	29655.8 ± 4040.2^b^
*Mb*	I	6.8 ± 0.8^a^	8.3 ± 0.6^ab^	10.2 ± 0.4^b^
*Mef2c*	I	1531.8 ± 336.6^a^	501.6 ± 129.1^b^	898.8 ± 69.1^a^
*Myh7*	I	35.6 ± 2.6^a^	42.1 ± 9.0^a^	85.6 ± 9.9^b^
*Ppargc1a*	I	50.5 ± 7.7^a^	136.8 ± 35.2^b^	118.1 ± 16.6^b^
*Tnnt1*	I	52.2 ± 3.9^a^	70.5 ± 21.0^a^	155.3 ± 16.1^b^
*Myh3*	Fetal or regenerating	1.3 ± 0.5^a^	0.7 ± 0.1^a^	39.1 ± 10.8^b^
*Myh8*	Perinatal or regenerating	5.2 ± 1.0^a^	4.0 ± 0.6^a^	12.2 ± 2.6^b^

*Actn3* alpha actinin 3, *Myh* myosin heavy chain, *Tnnt3* troponin T type 3, *Mb* myoglobin, *Mef2c* myocyte-specific enhancer factor 2C, *Ppargc1a* peroxisome proliferator-activated receptor gamma coactivator 1 alpha, *Tnnt1* troponin T type 1. Data = mean ± SEM (*n* = 4 or 5 mice). ^a,b,c^ Values not sharing the same *letter labels* are different for the same gene (*P* < 0.05).

### Postnatal *Stac3* deletion reduced muscle strength

**Fig. 9:**
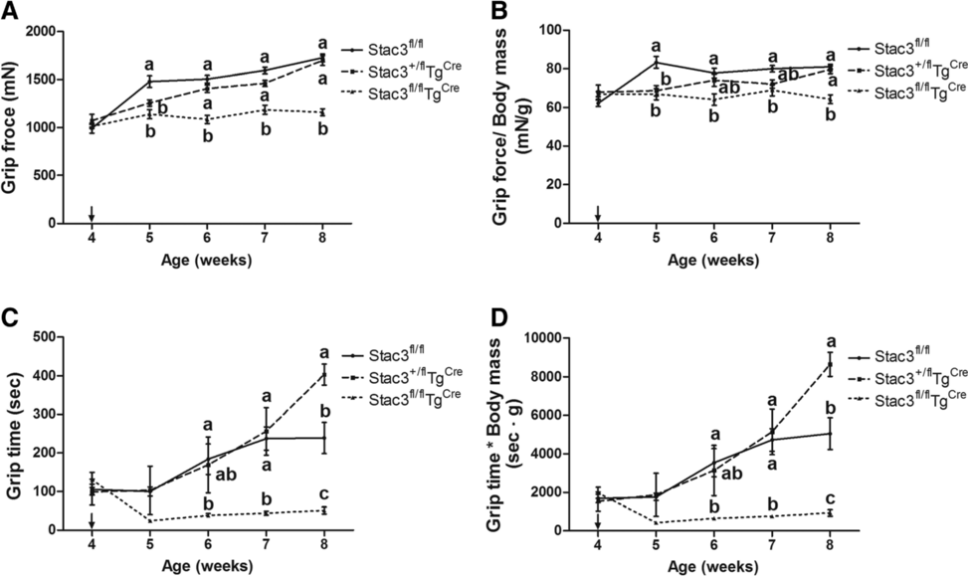
Muscle strength analysis of *Stac3* ^*fl/fl*^, *Stac3* ^*+/fl*^ *Tg* ^*Cre*^, and *Stac3* ^*fl/fl*^ *Tg* ^*Cre*^ mice before and after tamoxifen injection. Muscle strength was assessed by grip strength and grip time tests at 4, 5, 6, 7, and 8 weeks of age. Mice received tamoxifen injection on the first 5 days of week 5 of age. **a** Grip strength. **b** Grip strength normalized to body mass. **c** Grip time. **d** Grip time normalized to body mass. Values not sharing the same *letter labels* are different at the same age (*P* < 0.05, *n* = 4 mice)

*Stac3*^*fl/fl*^*Tg*^*Cre*^ mice had weaker grip strength than *Stac3*^*fl/fl*^ mice at each of the 4 weeks following tamoxifen injection (Fig. 9a, b, *P* < 0.05). *Stac3*^*+/fl*^*Tg*^*Cre*^ mice had weaker grip strength than *Stac3*^*fl/fl*^ mice at the first week after tamoxifen injection (Fig. 9a, b, *P* < 0.05). Compared to *Stac3*^*fl/fl*^ mice, *Stac3*^*fl/fl*^*Tg*^*Cre*^ mice had shorter grip time at the second, third, and fourth weeks after tamoxifen injection (Fig. 9c, d, *P* < 0.05). *Stac3*^*+/fl*^*Tg*^*Cre*^ mice had shorter grip time than *Stac3*^*fl/fl*^ mice at the fourth week after tamoxifen injection (Fig. 9a, b, *P* < 0.05). *Stac3*^*fl/fl*^*Tg*^*Cre*^, *Stac3*^*+/fl*^*Tg*^*Cre*^, and *Stac3*^*fl/fl*^ mice did not differ in grip strength or grip time at 4 weeks of age, i.e., before tamoxifen injection (Fig. 9a–d).

### Postnatal *Stac3* deletion reduced electrostimulation- but not caffeine-induced muscle contraction

**Fig. 10:**
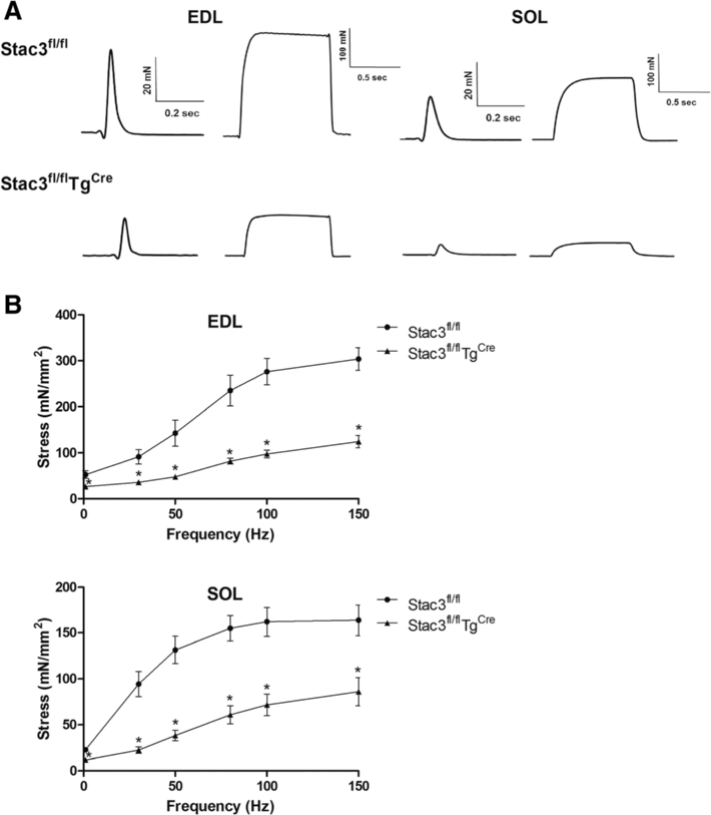
Electrostimulation-induced contractions in EDL and SOL muscles from tamoxifen-injected *Stac3* ^*fl/fl*^ and *Stac3* ^*fl/fl*^ *Tg* ^*Cre*^ mice at 8 weeks of age. **a** Representative twitch (1 Hz) and tetanic (150 Hz) contractile responses. **b** Stress-frequency relationships. Frequencies of electrostimulation used were 1, 30, 50, 80, 100, and 150 Hz. Force was normalized to the average cross-sectional area of the muscle to yield stress (mN/mm^2^). *Asterisks* indicate *P* < 0.05, *Stac3* ^*fl/fl*^ *Tg* ^*Cre*^ versus *Stac3* ^*fl/fl*^ at the same frequency (*n* = 4 mice for *Stac3* ^*fl/fl*^; *n* = 5 mice for *Stac3* ^*fl/fl*^ *Tg* ^*Cre*^)

We compared the contractile properties of the EDL and SOL muscles from *Stac3*^*fl/fl*^ and *Stac3*^*fl/fl*^*Tg*^*Cre*^ mice at 8 weeks of age, i.e., 4 weeks after tamoxifen injection. The stress-frequency responses to electrostimulation were more than 50 % smaller in the *Stac3*^*fl/fl*^*Tg*^*Cre*^ than in the *Stac3*^*fl/fl*^ EDL and SOL muscles (Fig. 10a, b, *P* < 0.05). However, for both twitch and tetanic (150 Hz) stresses, neither time to peak tension (TPT) nor half-relaxation time (HRT) was different between the *Stac3*^*fl/fl*^*Tg*^*Cre*^ and *Stac3*^*fl/fl*^ EDL or SOL muscle (Additional file 1: Table S2).

**Fig. 11:**
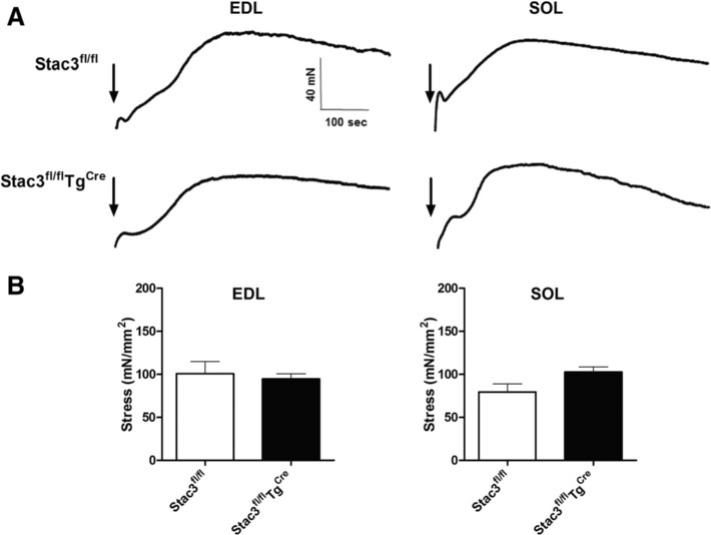
Caffeine-induced contractions in EDL and SOL muscles from tamoxifen-injected *Stac3* ^*fl/fl*^ and *Stac3* ^*fl/fl*^ *Tg* ^*Cre*^ mice at 8 weeks of age. The EDL and SOL muscles from mice 4 weeks after tamoxifen injection were stimulated by 25 mM caffeine. **a** Representative contractile responses. Black arrows indicate addition of caffeine. **b** Summary stress (mN/mm2) responses. Values are not different (*P* > 0.05) between *Stac3* ^*fl/fl*^ and *Stac3* ^*fl/fl*^ *Tg* ^*Cre*^ (n = 4 mice for *Stac3* ^*fl/fl*^; n = 5 mice for *Stac3* ^*fl/fl*^ *Tg* ^*Cre*^)

Caffeine induced both the EDL and SOL muscles from both 8-week-old *Stac3*^*fl/fl*^ and *Stac3*^*fl/fl*^*Tg*^*Cre*^ mice to contract (Fig. 11a). However, the caffeine-induced maximum tension in either muscle was not different between the two genotypes (Fig. 11b). The TPT for caffeine-induced contraction in either muscle was also not different between the two genotypes (Additional file 1: Table S2).

### Postnatal *Stac3* deletion reduced electrostimulation- but not caffeine-induced intracellular increase of calcium in single myofibers

**Fig. 12:**
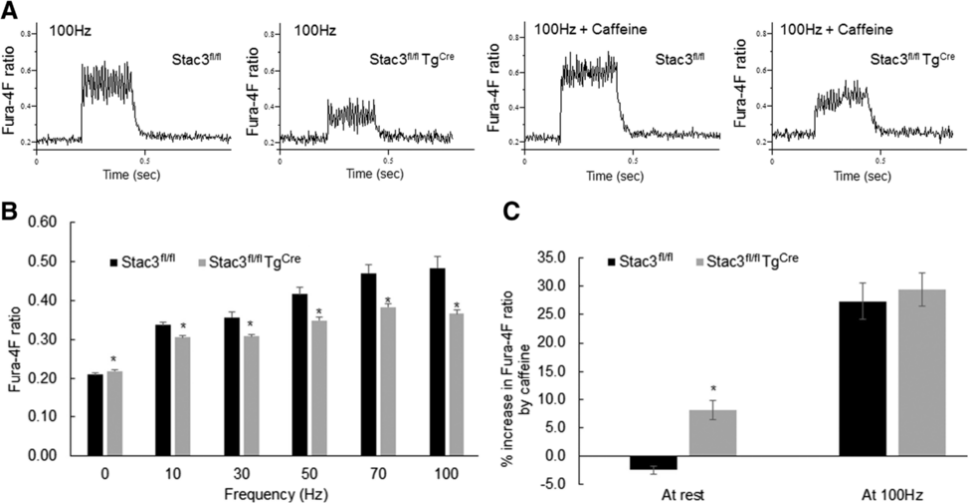
Electrostimulation- and caffeine-induced increases in intracellular calcium in FDB myofibers from tamoxifen-injected *Stac3* ^*fl/fl*^ and *Stac3* ^*fl/fl*^ *Tg* ^*Cre*^ mice at 8 weeks of age. The FDB myofibers from mice 4 weeks after tamoxifen injection were loaded with the fluorescent calcium indicator Fura-4F. Fluorescence emission was recorded by a microscope. **a** Representative raw Fura-4F traces. **b** Quantification of frequent electrostimulation-induced increases in intracellular calcium concentration. *Asterisks* indicate *P* < 0.05, *Stac3* ^*fl/fl*^ *Tg* ^*Cre*^ versus *Stac3* ^*fl/fl*^ at the same frequency (*n* = 37, 18 fibers for *Stac3* ^*fl/fl*^ *Tg* ^*Cre*^ and *Stac3* ^*fl/fl*^, respectively). **c** Quantification of caffeine-induced percentage changes in intracellular calcium concentration. *Asterisk* indicates *P* < 0.05, *Stac3* ^*fl/fl*^ *Tg* ^*Cre*^ versus *Stac3* ^*fl/fl*^

We compared electrostimulation-induced calcium release from the sarcoplasmic reticulum in single FDB myofibers from 8-week-old *Stac3*^*fl/fl*^ and *Stac3*^*fl/fl*^*Tg*^*Cre*^ mice (Fig. 12a; Additional file 6: Figure S5). Resting concentration of intracellular calcium was 4 % greater in *Stac3*^*fl/fl*^*Tg*^*Cre*^ than in *Stac3*^*fl/fl*^ myofibers (Fig. 12b, *P* < 0.05). Frequent electrostimulation increased the concentration of intracellular calcium in both *Stac3*^*fl/fl*^ and *Stac3*^*fl/fl*^*Tg*^*Cre*^ myofibers (Fig. 12b), but the increase was smaller in *Stac3*^*fl/fl*^*Tg*^*Cre*^ than in *Stac3*^*fl/fl*^ myofibers at each of the frequencies tested (Fig. 12b, *P* < 0.05; Additional file 6: Figure S5). Caffeine caused an 8 % increase in the concentration of intracellular calcium in resting *Stac3*^*fl/fl*^*Tg*^*Cre*^ myofibers, but it had no effect on that in resting *Stac3*^*fl/fl*^ myofibers (Fig. 12c, *P* < 0.05). Caffeine caused significant increases in the concentration of intracellular calcium in both *Stac3*^*fl/fl*^ and *Stac3*^*fl/fl*^*Tg*^*Cre*^ myofibers that were electrically stimulated (100 Hz), but these increases were not different between the two genotypes (Fig. 12c).

### Postnatal *Stac3* deletion reduced protein expression of RYR1 in skeletal muscle

**Fig. 13:**
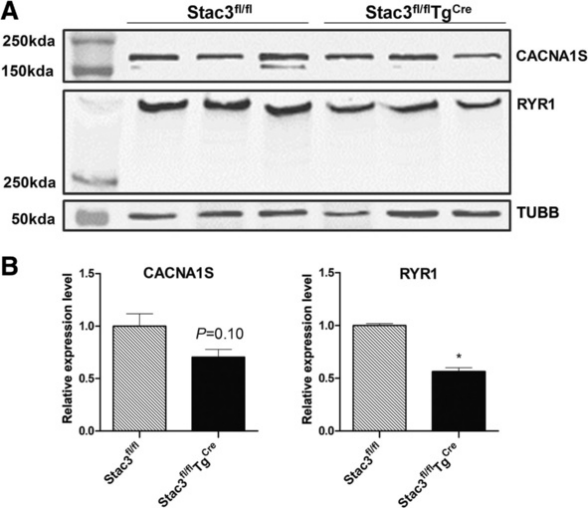
Western blotting analyses of CACNA1S and RYR1 in hindlimb muscles from tamoxifen-injected *Stac3* ^*fl/fl*^ and *Stac3* ^*fl/fl*^ *Tg* ^*Cre*^ mice at 8 weeks of age. **a** Representative western blots. Hindlimb muscles from three mice for each genotype were analyzed. Beta tubulin (TUBB) was detected as the loading control. Molecular markers are shown in the *first lane*. **b** Quantification of band intensities. Intensity of the CACNA1S or RYR1 band was normalized to that of the TUBB band in the same sample. *Asterisk*: *P* < 0.05, *Stac3* ^*fl/fl*^ *Tg* ^*Cre*^ versus *Stac3* ^*fl/fl*^

The DHPRα1 subunit CACNA1S (also known as Cav1.1) and RYR1 are essential for EC coupling. We compared the expression levels of these two proteins in hindlimb muscles from *Stac3*^*fl/fl*^*Tg*^*Cre*^ and *Stac3*^*fl/fl*^ mice at 8 weeks of age, i.e., 4 weeks after tamoxifen injection, by western blotting analyses. The expression level of RYR1 was nearly 50 % less in *Stac3*^*fl/fl*^*Tg*^*Cre*^ than in *Stac3*^*fl/fl*^*Tg*^*Cre*^ mice (Fig. 13, *P* < 0.05). The expression level of CACNA1S was 30 % less in *Stac3*^*fl/fl*^*Tg*^*Cre*^ than in *Stac3*^*fl/fl*^*Tg*^*Cre*^ mice (Fig. 13, *P* = 0.10).

## Discussion

In this study, we characterized the effects of postnatal *Stac3* deletion on skeletal muscle growth, fiber composition, and contractile responses in mice. We deleted the *Stac3* gene in postnatal mice through the Flp-FRT and tamoxifen-inducible Cre-loxP recombination systems [8, 9]. At the mRNA level, *Stac3* expression in skeletal muscle was reduced to nearly none 4 weeks after tamoxifen injection. This low expression indicates that the floxed *Stac3* gene was efficiently excised by tamoxifen-induced Cre recombinase in skeletal muscle. However, at the protein level, STAC3 expression in skeletal muscle was reduced by 70 % 4 weeks after tamoxifen injection, which was less than that observed for *Stac3* mRNA. This discrepancy was perhaps caused by the STAC3 protein being very stable or the translation of *Stac3* mRNA into STAC3 protein being very efficient in mouse skeletal muscle. Nevertheless, the phenotypic changes in *Stac3*-deleted mice suggest that a 70 % reduction in STAC3 protein expression is sufficient to alter the functions of this protein in mouse skeletal muscle.

Our data showed that postnatal *Stac3* deletion caused several significant phenotypic changes in skeletal muscle. One change was reduced skeletal muscle growth. Histological analyses showed that the skeletal muscle of *Stac3*-deleted mice had a smaller CSA but a similar number of myofibers compared to that of control mice. These data suggest that STAC3 is needed for normal myofiber hypertrophy in postnatal mice. Myofiber hypertrophy results from increased protein synthesis and reduced protein degradation. The IGF1-IGFIR-PI3K-Akt/PKB-mTOR and the myostatin-ACTRII-Smad2/3 are two primary signaling pathways that regulate protein accumulation, positively and negatively, respectively, in the skeletal muscle [18, 19]. It remains to be determined if *Stac3* deletion inhibits muscle hypertrophy by influencing these signaling pathways.

The second phenotypic change in *Stac3*-deleted muscle was an increased number of myofibers containing centralized nuclei. The histological analyses indicated that these centrally nucleated myofibers were among the smallest fibers in the muscles analyzed. *Stac3*-deleted skeletal muscle had an increased expression of *Myh3* and *Myh8* mRNAs. Since these two myosin heavy chain genes are typically expressed in fetal and neonatal muscle or regenerating adult muscle [16, 17], the increased myofibers with centralized nuclei in 8-week-old *Stac3*-deleted mice represent either those from the fetal or neonatal stage or regenerating myofibers in adult muscle. Interestingly, embryonic *Stac3* deletion caused the majority of myofibers in newborn mice to have centralized nuclei [1]. It remains to be determined if the increased myofibers with centralized nuclei in 8-week-old *Stac3*-deleted mice result from retarded myofiber growth or myofiber regeneration.

The third phenotypic change caused by postnatal *Stac3* deletion was a muscle fiber-type switch. Postnatal *Stac3* deletion increased the percentage of type I fibers in the fast twitch EDL muscle and the oxidative capacity in the slow twitch SOL muscle. Since *Stac3* deletion did not alter the total number of myofibers in these muscles, the increase in type I or oxidative fibers suggests that *Stac3* deletion inhibited the type I to type II transition in fast twitch muscle while stimulating the glycolytic to oxidative fiber transition in slow twitch muscle. Such an effect of *Stac3* deletion on fiber-type switch is supported by the facts that *Stac3*-deleted limb muscles in general had greater expression of genes marking type I fibers and less expression of genes marking type II fibers than control limb muscles and that *Stac3*-deleted muscles appeared redder and had greater expression of myoglobin mRNA than control muscles.

The fourth phenotypic change caused by postnatal *Stac3* deletion in mice was reduced muscle force production. Both grip strength and grip time tests indicated that beginning the first week after tamoxifen injection, muscle strength was reduced in *Stac3*-deleted mice and that this reduction was not recovered 4 weeks after tamoxifen injection. Both grip strength and grip time in *Stac3*-deleted mice were less than those in control mice after being normalized to body mass. This data suggests the *Stac3* deletion-caused reduction in muscle grip strength was not due to reduction in muscle size. Muscle contractile function tests showed that electrostimulation-induced maximum stress was smaller in *Stac3*-deleted compared to control muscle. Because stress was muscle force normalized to CSA, this data further suggested that the decreased muscle size was not responsible for the reduced contractile forces.

In this study, we found that postnatal *Stac3* deletion reduced electrostimulation- but not caffeine-induced muscle contractions and calcium release from the sarcoplasmic reticulum (SR). This finding is consistent with earlier studies in *Stac3*-deleted mouse fetuses and *Stac3*-mutated zebrafish [2, 3]. In this study, we found that the total cellular expression of RYR1 and, to a lesser degree, that of CACNA1S, were reduced in skeletal muscle of *Stac3*-deleted postnatal mice compared to control mice. Reduced expressions of CACNA1S and RYR1 were also observed in the skeletal muscle of *Stac3*-deleted fetal mice [2]. These observations suggest that *Stac3* deletion might affect electrostimulation-induced calcium release from the SR and contraction in part by reducing the expression and/or the stability of RYR1 and CACNA1S proteins in the skeletal muscle. A recent study by Polster et al. demonstrates that STAC3 can target the DHPRα1 subunit CACNA1S to the plasma membrane of tsA201 cells [4]. In this study, we did not determine if *Stac3*-deleted skeletal muscle lacked CACNA1S on the sarcolemma or T-tubules, but a T-tubular deficiency of CACNA1S would explain why *Stac3*-deleted muscle was deficient in electrostimulation-induced calcium release from the SR and contraction. A sarcolemma-specific reduction of CACNA1S expression in adult mice has been shown to cause muscle atrophy without affecting EC coupling [20]. Thus, a sarcolemmal deficiency of CACNA1S expression would explain our observation that *Stac3* deletion inhibited muscle hypertrophy in young mice. The attenuated muscle stress responses in *Stac3*-deleted muscle to electrostimulation could be also due to defective membrane depolarization, defective voltage-dependent activation of DHPR, and/or deficient DHPR-RYR1 coupling. These possibilities remain to be characterized in the future. In this study, we also found that *Stac3*-deleted myofibers had a greater release of calcium from the SR in response to the ryanodine receptor agonist caffeine compared to control myofibers. This result suggests that *Stac3* deletion might increase the sensitivity of RYR1 to caffeine or the amount of releasable calcium in the SR.

Muscle activity can affect both muscle size and fiber type [21, 22]; thus, it is tempting to speculate that *Stac3* deletion-induced changes in fiber size and type were secondary to changed muscle activity. Increased muscle activity generally contributes to fiber hypertrophy, whereas reduced muscle activity could result in muscle atrophy, perhaps through the calcium/calcineurin signaling pathway [23, 24]. As such, it is possible that *Stac3* deletion-reduced muscle contraction is responsible for reduced fiber growth. However, the same mechanism may not be responsible for *Stac3* deletion-caused type II to type I fiber transformation because reduced muscle activity usually leads to type I to type II fiber switch [25]. The STAC3 protein contains a SH3 domain, and through this domain, STAC3 could interact with and affect the expression, location, or function of many proteins [26]. Therefore, it is possible that STAC3 mediates EC coupling, fiber-type switch, and fiber hypertrophy through different protein partners.

## Conclusions

This study demonstrates that *Stac3* is important to fiber growth, fiber-type composition, contractile responses, and electrostimulation-induced release of calcium ions from the sarcoplasmic reticulum in postnatal skeletal muscle and that the role of STAC3 in skeletal muscle may be beyond the process of EC coupling. Since *Stac3* knockout is embryonically lethal, the postnatal *Stac3* knockout mouse generated in the present study will be a more convenient model for understanding the role of STAC3 in skeletal muscle physiology and disease and the underlying cellular and molecular mechanisms.

## Additional files

Additional file 1: Table S1. Nucleotide sequences of primers used in this study. Table S2. Contractile properties of EDL and SOL muscles from 8-week-old *Stac3* ^*fl/fl*^ and *Stac3* ^*fl/fl*^ *Tg* ^*Cre*^ mice. (DOCX 18 kb) Additional file 2: Figure S1. Histological analysis of EDL muscles from 4-week-old *Stac3* ^*fl/fl*^, *Stac3* ^*+/fl*^ *Tg* ^*Cre*^, and *Stac3* ^*fl/fl*^ *Tg* ^*Cre*^ mice. Cross-sections of EDL muscles were stained with hematoxylin and eosin. **a** Representative images of stained EDL sections at ×40 and ×400 magnifications. *Scale bars*: 100 μm for ×40 and 10 μm for ×400. *Arrows* point to myofibers with centralized nuclei. **b** Average number of myofibers per cross-section. This number was not different between genotypes. **c** Average cross-sectional area (CSA) of myofibers. **d** Distribution of CSA of myofibers. **e** Percentage of myofibers containing centralized nuclei. (TIF 74102 kb) Additional file 3: Figure S2. Histological analysis of SOL muscles from 4-week-old *Stac3* ^*fl/fl*^, *Stac3* ^*+/fl*^ *Tg* ^*Cre*^, and *Stac3* ^*fl/fl*^ *Tg* ^*Cre*^ mice. Cross-sections of SOL muscles were stained with hematoxylin and eosin. **a** Representative images of stained SOL sections at ×40 and ×400 magnifications. *Scale bars*: 100 μm for ×40 and 10 μm for ×400. *Arrows* point to myofibers with centrally located nuclei. **b** Average number of myofibers per cross-section. This number was not different between genotypes. **c** Average cross-sectional area (CSA) of myofibers. **d** Distribution of CSA of myofibers. **e** Percentage of myofibers containing centralized nuclei. (TIF 73696 kb) Additional file 4: Figure S3. Myosin-ATPase staining (pH 4.21) of cross-sections of EDL and SOL muscles from 4-week-old *Stac3* ^*fl/fl*^, *Stac3* ^*+/fl*^ *Tg* ^*Cre*^, and *Stac3* ^*fl/fl*^ *Tg* ^*Cre*^ mice before tamoxifen injection. **a** Representative images of stained muscle sections. *Scale bars*: 100 μm. **b** Percentages of type I and type II myofibers in EDL muscles. **c** Percentages of type I and type II myofibers in SOL muscles. None of the percentages are different between genotypes (*n* = 4 mice). (TIF 50091 kb) Additional file 5: Figure S4. NADH-TR staining of cross-sections of EDL and SOL muscles from 4-week-old *Stac3* ^*fl/fl*^, *Stac3* ^*+/fl*^ *Tg* ^*Cre*^, and *Stac3* ^*fl/fl*^ *Tg* ^*Cre*^ mice before tamoxifen injection. **a** Representative images of NADH-TR staining. *Scale bars*: 100 μm. **b** Percentages of light-stained and dark-stained myofibers in EDL muscles. **c** Percentages of light-stained and dark-stained myofibers in SOL muscles. None of the percentages are different between genotypes (*n* = 4 mice). (TIF 51536 kb) Additional file 6: Figure S5. Quantification of electrostimulation-induced increases in intracellular calcium concentration in FDB myofibers from tamoxifen-injected *Stac3* ^*fl/fl*^ and *Stac3* ^*fl/fl*^ *Tg* ^*Cre*^ mice at 8 weeks of age. The FDB myofibers from mice 4 weeks after tamoxifen injection were loaded with the fluorescent calcium indicator Fura-2. Fluorescence emission was recorded by a microscope. *Asterisks* indicate *P* < 0.05, *Stac3* ^*fl/fl*^ *Tg* ^*Cre*^ versus *Stac3* ^*fl/fl*^ at the same frequency (*n* = 8 fibers each genotype). (TIF 9896 kb)

## Abbreviations

*Actn3*: alpha actinin 3
CACNA1S (also known as Cav1.1): calcium channel, voltage-dependent, L-type, alpha 1S subunit
CSA: cross-sectional area
DHPR: dihydropyridine receptor
DHPRα1s: dihydropyridine receptor, alpha 1S subunit
EC: excitation-contraction
EDL: extensor digitorum longus
FBS: fetal bovine serum
FDB: flexor digitorum brevis
Flp: flippase
FRT: flippase recognition target
HRT: half-relaxation time
*Mb*: myoglobin
*Mef2c*: myocyte-specific enhancer factor 2C
mTOR: mammalian target of rapamycin
*Myh*: myosin heavy chain
NADH-TR: nicotinamide adenine dinucleotide-tetrazolium reductase
*Ppargc1a*: peroxisome proliferator-activated receptor gamma coactivator 1 alpha
PSS: physiological salt solution
RIPA: radio immunoprecipitation assay
RT-qPCR: reverse transcription-quantitative polymerase chain reaction
RYR: ryanodine receptor
SOL: soleus
*Stac3*: SH3 and cysteine-rich domain 3
TA: tibialis anterior
*Tnnt1*: troponin T type 1
*Tnnt3*: troponin T type 3
TPT: time to peak tension
TUBB: beta tubulin
