# Supplementary material for: The SH3 and cysteine-rich domain 3 (Stac3) gene is important to growth, fiber composition, and calcium release from the sarcoplasmic reticulum in postnatal skeletal muscle
Source: Skelet Muscle. 2016 Apr 11;6:17. doi: 10.1186/s13395-016-0088-4 (PMC4828897; doi:10.1186/s13395-016-0088-4)
Supplement: Additional file 1: Table S1. — Nucleotide sequences of primers used in this study. Table S2. Contractile properties of EDL and SOL muscles from 8-week-old Stac3 fl/fl and Stac3 fl/fl Tg Cre mice. (DOCX 18 kb) [file 13395_2016_88_MOESM1_ESM.docx]

**Additional file 1**

**Table S1. Nucleotide sequences of primers used in this study.**

| Gene | Direction | Primer sequence | GenBank  Accession # |
| --- | --- | --- | --- |
| *Actn3* | Forward  Reverse | 5’- ATATCGTGAACACCCCCAAA -3’  5’- TCCACTCCAACAGCTCACTG -3’ | NM_013456 |
| *Cre*  *Flp*  *Mb* | Forward  Reverse  Forward  Reverse  Forward  Reverse | 5’- GCGGTCTGCAGTAAAAACTATC -3’  5’- GTGAAACAGCATTGCTGTCACTT -3’  5’- CACTGATATTGTAAGTAGTTTGC -3’  5’- CTAGTGCGAAGTAGTGATCAGG -3’  5’- ATGTGAGGGCCAGAGAAAGG -3’  5’- TCCAGGTACTTGACCGGGAT -3’ | NM_001164047 |
| *Mef2c* | Forward  Reverse | 5’- AGAAGTGCAGAGGGAACGAA -3’  5’- CGCTCATCCATTATCCTCGT -3’ | NM_001170537 |
| *Myog* | Forward  Reverse | 5’- CGGCTGCCTAAAGTGGAGAT -3’  5’- AGGCCTGTAGGCGCTCAA -3’ | NM_031189 |
| *Myh1* | Forward  Reverse | 5’- AGTCCCAGGTCAACAAGCTG -3’  5’- CACATTTGCTCATCTTTGG -3’ | NM_030679 |
| *Myh2* | Forward  Reverse | 5’- AGTCCCAGGTCAACAAGCTG -3’  5’- GCATGACCAAAGGTTTCACA -3’ | NM_001039545 |
| *Myh3* | Forward  Reverse | 5’- CGCAGAATCGCAAGTCAATA -3’  5’- ATATCTTCTGCCCTGCACCA -3’ | NM_001099635 |
| *Myh4* | Forward  Reverse | 5’- AGTCCCAGGTCAACAAGCTG -3’  5’- TTTCTCCTGTCACCTCTCAACA -3’ | NM_010855 |
| *Myh7* | Forward  Reverse | 5’- AGTCCCAGGTCAACAAGCTG -3’  5’- TTCCACCTAAAGGGCTGTTC -3’ | NM_080728 |
| *Myh8* | Forward  Reverse | 5’- AGTCCCAGGTCAACAAGCTG -3’  5’- CCTCCTGTGCTTTCCTTCAG -3’ | NM_177369 |
| *Ppargc1a* | Forward  Reverse | 5’- AATGCAGCGGTCTTAGCACT -3’  5’- TTTCTGTGGGTTTGGTGTGA -3’ | NM_008904 |
| *Rn18s* | Forward  Reverse | 5’- TTAAGAGGGACGGCCGGGGG -3’  5’- CTCTGGTCCGTCTTGCGCCG -3’ | NM_003278 |
| *Stac3*  *Stac3*  *Tnnt1* | F1  R1  F2  R2  Forward  Reverse | 5’- CTCCATAGCTCTACCGCAGTC -3’  5’- CTCTGCCTTGTGAGTGTGGA -3’  5’- GAATGGAGGGGTAAGGGAAG -3’  5’- ACCCACAGGTTGAGAACAGC -3’  5’- AAACCCAGCCGTCCTGTG -3’  5’- TCATCTCCCGACCAGTCTGT -3’ | JN959041.1  JN959041.1  NM_001277903 |
| *Tnnt3* | Forward  Reverse | 5’- GCCCAAGAGGAAGAAGTCCA -3’  5’- TAGCTGCTGTAGTTGGCACC -3’ | NR_001163664 |

*Actn3*, alpha actinin 3; *Cre*, Cre recombinase coding gene; *Flp*, Flp recombinase coding gene; *Mb*, myoglobin; *Mef2c*, myocyte specific enhancer factor 2C; *Myog*, myogenin; *Myh*, myosin heavy chain; *Ppargc1a*, peroxisome proliferator-activated receptor gamma coactivator 1 alpha; *Rn18s*, 18S ribosomal RNA; *Tnnt1*, troponin T type 1; *Tnnt3*, troponin T type 3.

**Table S2. Contractile properties of EDL and SOL muscles from 8-week-old Stac3^fl/fl^ and Stac3^fl/fl^Tg^Cre^ mice.**

|  |  | | EDL | | | | SOL | | |
| --- | --- | --- | --- | --- | --- | --- | --- | --- | --- |
|  |  | | Stac3^fl/fl^  (n=4) | | Stac3^fl/fl^Tg^Cre^ (n=5) | | | Stac3^fl/fl^  (n=4) | Stac3^fl/fl^Tg^Cre^ (n=5) |
| Twitch | | Stress (mN/mm^2^) | | 52.6 ± 8.1 | | 26.5 ± 2.4^*^ | | 23.0 ± 2.8 | 12.2 ± 2.6* |
|  | | TPT (ms) | | 19.9 ± 0.4 | | 18.6 ± 0.9 | | 29.6 ± 0.7 | 22.1 ± 1.0 |
|  | | HRT (ms) | | 17.1 ± 1.7 | | 15.7 ± 2.1 | | 34.4 ± 5.4 | 23.8 ± 2.1 |
| Tetanus | | Stress (mN/mm^2^) | | 303.8 ± 24.7 | | 124.3 ± 13.6^*^ | | 163.8 ± 17.0 | 86.3 ± 15.1* |
|  | | TPT (ms) | | 320.0 ± 77.1 | | 512.0 ± 81.8 | | 637.0 ± 24.6 | 765.9 ± 68.4 |
|  | | HRT (ms) | | 556.0 ± 107.9 | | 325.4 ± 75.4 | | 200.3 ± 51.8 | 104.0 ± 44.4 |
| Caffeine^#^ | | Stress (mN/mm^2^) | | 100.9 ± 14.4 | | 94.7 ± 6.1 | | 79.6 ± 9.3 | 102.8 ± 6.0 |
|  | | TPT (ms) | | 281.8 ± 16.8 | | 280.2 ± 26.3 | | 214.8 ± 12.0 | 176.2 ± 13.9 |

Stress, force/cross-sectional area. TPT, time to peak tension. HRT, half relaxation time. Data are presented as mean ± SEM. * indicates *P* < 0.05, *Stac3^fl/fl^Tg^Cre^ vs. Stac3^fl/fl^* within EDL or SOL. ^#^HRT for caffeine-induced contraction could not be determined because recording was stopped before muscle completely relaxed.
