# Supplementary figures and images for: The SH3 and cysteine-rich domain 3 (Stac3) gene is important to growth, fiber composition, and calcium release from the sarcoplasmic reticulum in postnatal skeletal muscle
Source: Skelet Muscle. 2016 Apr 11;6:17. doi: 10.1186/s13395-016-0088-4 (PMC4828897; doi:10.1186/s13395-016-0088-4)

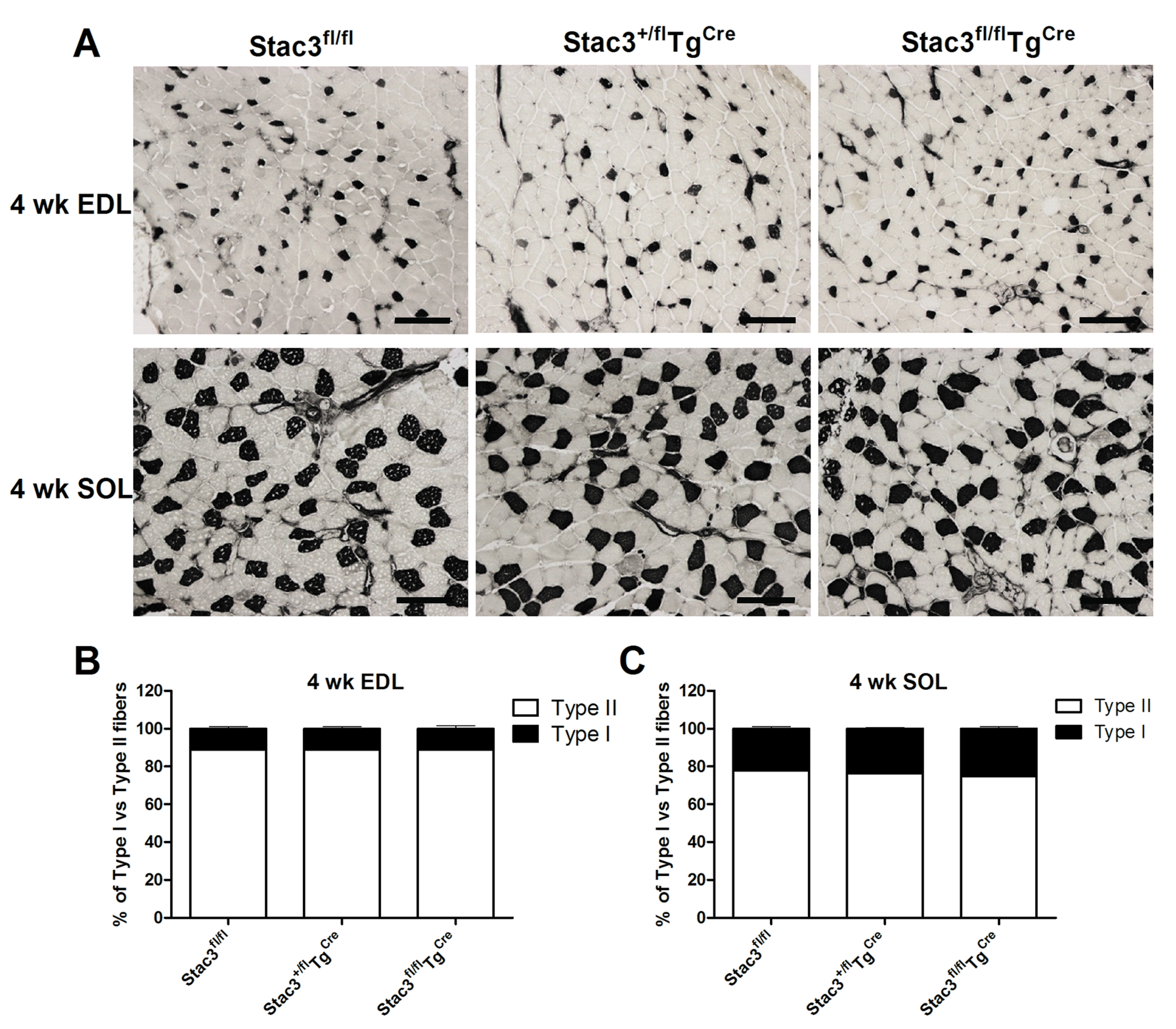

Supplement: Additional file 4: Figure S3. — Myosin-ATPase staining (pH 4.21) of cross-sections of EDL and SOL muscles from 4-week-old Stac3 fl/fl, Stac3 +/fl Tg Cre, and Stac3 fl/fl Tg Cre mice before tamoxifen injection. a Representative images of stained muscle sections. Scale bars: 100 μm. b Percentages of type I and type II myofibers in EDL muscles. c Percentages of type I and type II myofibers in SOL muscles. None of the percentages are different between genotypes (n = 4 mice). (TIF 50091 kb) [file 13395_2016_88_MOESM4_ESM.tif]

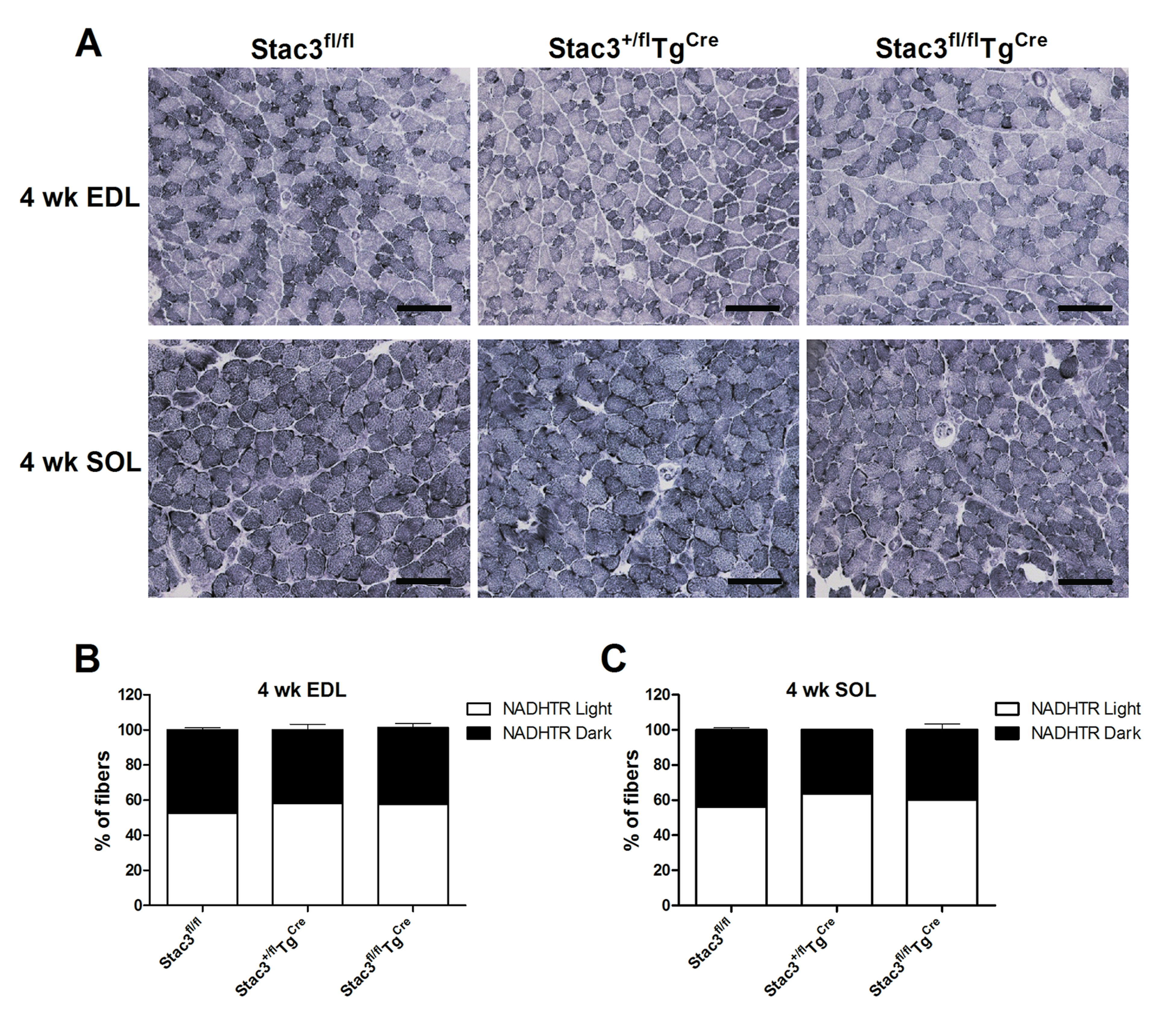

Supplement: Additional file 5: Figure S4. — NADH-TR staining of cross-sections of EDL and SOL muscles from 4-week-old Stac3 fl/fl, Stac3 +/fl Tg Cre, and Stac3 fl/fl Tg Cre mice before tamoxifen injection. a Representative images of NADH-TR staining. Scale bars: 100 μm. b Percentages of light-stained and dark-stained myofibers in EDL muscles. c Percentages of light-stained and dark-stained myofibers in SOL muscles. None of the percentages are different between genotypes (n = 4 mice). (TIF 51536 kb) [file 13395_2016_88_MOESM5_ESM.tif]

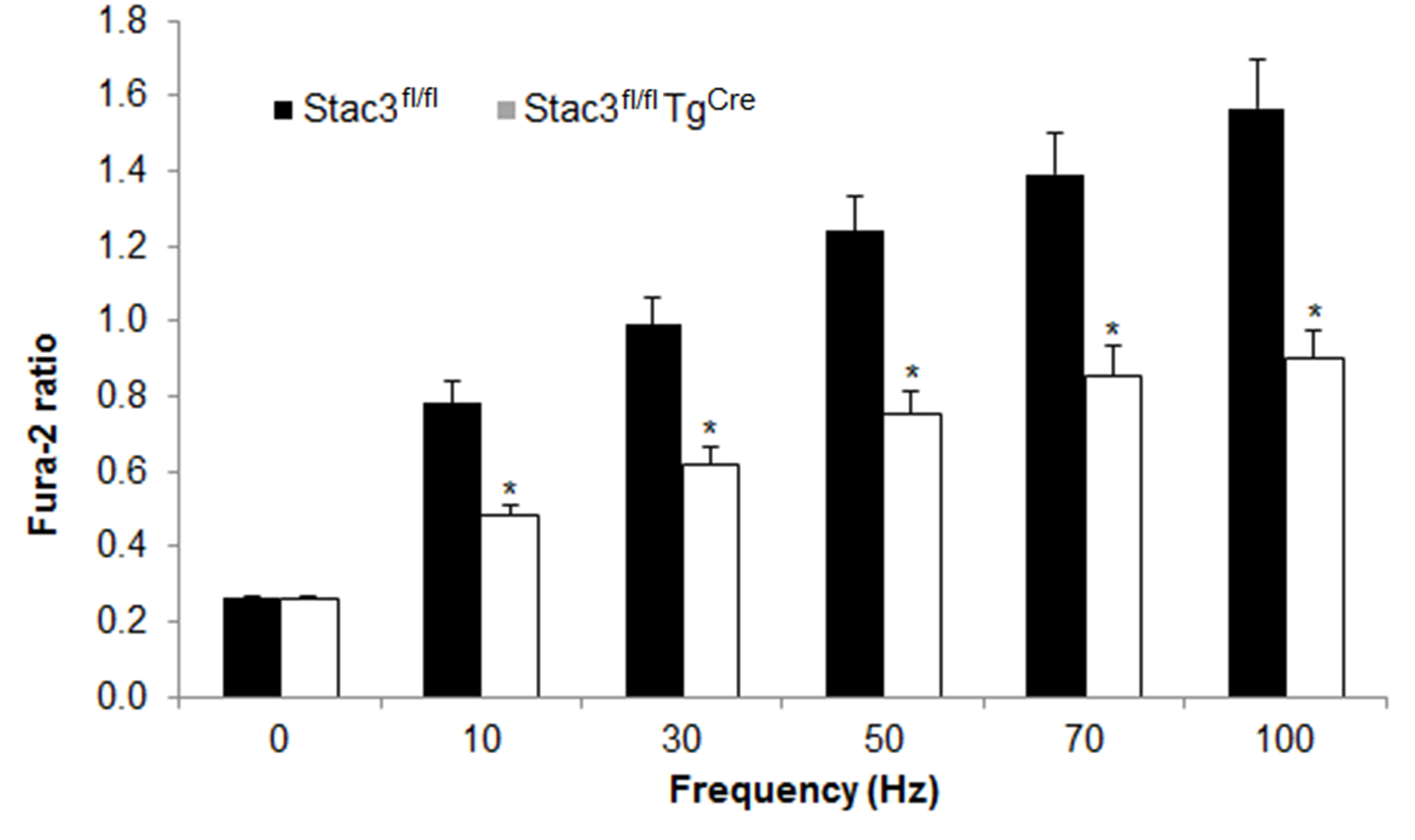

Supplement: Additional file 6: Figure S5. — Quantification of electrostimulation-induced increases in intracellular calcium concentration in FDB myofibers from tamoxifen-injected Stac3 fl/fl and Stac3 fl/fl Tg Cre mice at 8 weeks of age. The FDB myofibers from mice 4 weeks after tamoxifen injection were loaded with the fluorescent calcium indicator Fura-2. Fluorescence emission was recorded by a microscope. Asterisks indicate P < 0.05, Stac3 fl/fl Tg Cre versus Stac3 fl/fl at the same frequency (n = 8 fibers each genotype). (TIF 9896 kb) [file 13395_2016_88_MOESM6_ESM.tif]
